# Wearable Technology Applications and Methods to Assess Clinical Outcomes in Foot and Ankle Disorders: Achievements and Perspectives

**DOI:** 10.3390/s24217059

**Published:** 2024-11-01

**Authors:** Lorenzo Brognara, Antonio Mazzotti, Simone Ottavio Zielli, Alberto Arceri, Elena Artioli, Francesco Traina, Cesare Faldini

**Affiliations:** 1 Department of Biomedical and Neuromotor Sciences (DIBINEM), University of Bologna, 40127 Bologna, Italy; lorenzo.brognara2@unibo.it; 2 1st Orthopaedics and Traumatologic Clinic, IRCCS Istituto Ortopedico Rizzoli, 40136 Bologna, Italy; simoneottavio.zielli@ior.it (S.O.Z.); alberto.arceri@ior.it (A.A.); elena.artioli@ior.it (E.A.); francesco.traina@ior.it (F.T.); cesare.faldini@ior.it (C.F.); 3 Department of Biomedical and Neuromotor Sciences (DIBINEM), Alma Mater Studiorum University of Bologna, 40126 Bologna, Italy

**Keywords:** wearable sensor, accelerometer, outcome assessment

## Abstract

Foot and ankle disorders are a very common diseases, represent a risk factor for falls in older people, and are associated with difficulty performing activities of daily living. With an increasing demand for cost-effective and high-quality clinical services, wearable technology can be strategic in extending our reach to patients with foot and ankle disorders. In recent years, wearable sensors have been increasingly utilized to assess the clinical outcomes of surgery, rehabilitation, and orthotic treatments. This article highlights recent achievements and developments in wearable sensor-based foot and ankle clinical assessment. An increasing number of studies have established the feasibility and effectiveness of wearable technology tools for foot and ankle disorders. Different methods and outcomes for feasibility studies have been introduced, such as satisfaction and efficacy in rehabilitation, surgical, and orthotic treatments. Currently, the widespread application of wearable sensors in clinical fields is hindered by a lack of robust evidence; in fact, only a few tests and analysis protocols are validated with cut-off values reported in the literature. However, nowadays, these tools are useful in quantifying clinical results before and after clinical treatments, providing useful data, also collected in real-life conditions, on the results of therapies.

## 1. Introduction

Foot and ankle disorders are very common in the general population, often resulting in walking difficulties, impaired physical functioning, and motor and mobility limitations, which significantly reduce the quality of life [1,2,3,4,5]. Various healthcare professionals, including orthopedic surgeons, physiatrists, rheumatologists, podiatrists, and physical therapists, are involved in the diagnosis, treatment, and rehabilitation of these patients. The therapeutic options available for these conditions range from conservative approaches, such as orthotics, rehabilitation, and physical therapies, to surgical interventions [6,7].

Traditionally, physicians diagnose and evaluate the response to treatments of these impairments through physical examinations, along with the use of clinical scoring systems and staging scales [8]. Among the assessment tools available, gait analysis is currently considered one of the most precise methods for evaluating various pathological conditions [9]. This is due to its objectivity and ability to measure parameters that are difficult to assess with conventional orthopedic examinations. Furthermore, gait analysis plays a crucial role in evaluating post-treatment outcomes, providing objective data to measure the effectiveness of one or more therapies [10]. This, in turn, helps healthcare professionals select the most appropriate therapeutic approach for each individual patient. However, gait analysis remains a costly tool available in a limited number of hospitals, making it accessible to only a small fraction of patients [11].

In this context, wearable technologies (WT) offer a significant opportunity for the collection of new clinical endpoints in clinical settings. WT can support and validate current therapies or help in the development of new clinical indices. The recent advancements in WT, including smartwatches, smartphones, smart glasses, and smart insoles, along with their increasing affordability, provide the ability to continuously collect large amounts of data outside the traditional laboratory environment [12]. Advances in ergonomics have made these tools smaller, lighter, and more powerful [13,14,15].

WT have the potential to assist clinicians using gait analysis not only in the diagnostic phase but also in the quantitative assessment of outcomes by measuring the reestablishment of function in daily life, thus evaluating the success of the therapy [16,17]. There is a growing body of literature that highlights the increasing interest in this technology, which promises to become a valuable tool for clinicians.

The aim of this article is to provide a comprehensive review of the various gait analysis methods using WT currently reported in the literature, focusing on (1) outcome variables, (2) data collection methods, (3) different types of WT available, and (4) barriers and limitations.

## 2. Outcome Variables

Traditionally, variables collected during a physical examination in a hospital setting are numerous, but they share certain common characteristics: these tests are typically brief, rarely exceeding 10 min, and are conducted on standard surfaces or steps or use various supports to evaluate movements such as transitioning from sitting to standing. While these tests are valuable, they have limitations, particularly in their inability to account for the real-world challenges that patients encounter outside the clinical environment.

An example of this limitation in current clinical approaches, as highlighted in other areas, is the difficulty in diagnosing and objectively correlating the functional instability of a joint with its laxity when assessed using different methods in an outpatient setting [18]. This is partly because these tests are often conducted with the patient in a static position—either lying down or sitting—far removed from the dynamic situations where instability manifests. Additionally, this approach excludes the evaluation of secondary stabilizers, such as muscular strength, neuromuscular control, and capsuloligamentous laxity, which are critical for maintaining joint stability during dynamic activities.

**Figure 1:**
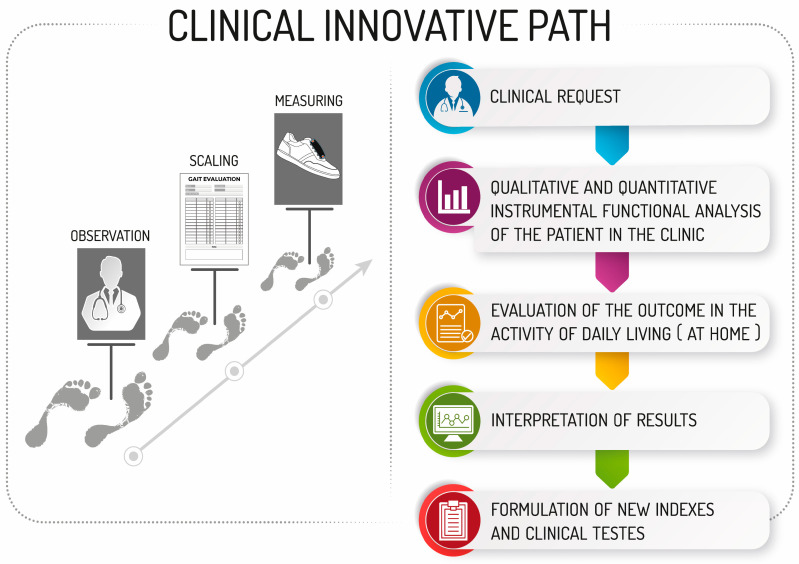
Clinical innovative path.

In this context, the emergence and widespread use of wearable devices, which can potentially be worn by the patient 24 h a day, allow for the expansion of these evaluations into activities outside the clinical setting and in a manner that is highly personalized for each patient. The potential of these devices lies in both supporting and validating the scores currently collected in clinical settings and in helping to gather data that could be used to develop new indices for a more quantitative and qualitative assessment of a specific therapy (Figure 1).

Wearable sensors, in particular inertial measurement units (IMUs), allow the objective assessment of quantitative outcomes during functional tests, some of which are already validated, such as the timed up and go test [19]. Good reliability has been seen in the literature in healthy patients, patients with Parkinson’s disease, older adults at risk of falling, and cognitively impaired patients [19,20,21,22,23]. Some spatiotemporal parameters, such as gait velocity, step length, and step time recorded during the timed up and go test and 6 min walk test with IMUs, were significantly correlated with some crucial scales such as the Expanded Disability Status Scale (EDSS) score, the Multiple Sclerosis Walking Scale-12 score, the revised amyotrophic lateral sclerosis functional rating scale (ALSFRS-R), and the Addenbrooke’s Cognitive Examination Revised (ACE-RACE-R score) [13,22,24].

## 3. Assessment of Foot and Ankle Joint Range of Motion

The measurement of foot and ankle joint range of motion (ROM) is a critical indicator of joint function and is commonly used to assess and quantify a patient’s progress in a rehabilitation program or to identify limitations in joint movement. Traditionally, ROM has been evaluated using a manual goniometer, which has limitations due to the challenge of consistently locating anatomical landmarks across different individuals and the difficulty of measuring ROM during activities that truly test these variables, such as walking, jumping, or running.

In recent decades, new digital instruments for measuring ROM have been developed based on various technologies: gyroscopes, which provide angular velocity around three orthogonal axes; magnetometers, which measure orientation relative to the Earth’s magnetic field; and accelerometers [25,26]. The use of wearable technology for measuring joint angles was developed to overcome the limitations of the manual universal goniometer, which is considered time-consuming and challenging to use for obtaining repeatable measurements [27,28]. Alongside the development of these technologies, alternative types of goniometers have emerged in clinical practice, incorporating various technologies such as digital inclinometers or even mobile apps, which, although potentially having a higher margin of error, offer the advantage of easy accessibility and availability while also demonstrating good reliability [29,30,31,32].

### 3.1. Assessment of Gait and Posture in People with Foot and Ankle Disorders

Foot and ankle problems, particularly foot pain, can impair balance and functional ability and have been associated with frailty level [33,34]. Routinely assessing and managing foot problems using wearable technology holds the potential to enable the early detection of issues and identify deteriorations in balance and physical activity. This early intervention could help preserve motor function by alleviating the fear of falling among patients with foot and ankle conditions. Ultimately, this approach may contribute to healthier aging and a reduced susceptibility to frailty [35].

Balance is a crucial parameter for identifying individuals at risk of falls or injury and can be assessed using various tools. Wearable devices have shown moderate to good intra-trial reliability and a strong correlation with postural sway measures on a force plate, which is considered the gold standard. IMUs can provide valuable insights for targeted interventions [36], with observations made in both the medio-lateral and antero-posterior directions whether the eyes are open or closed [37]. Foot orthoses have demonstrated effectiveness in improving balance and reducing pain and disability in elderly women with osteoporosis [38], serving as an adjunctive approach to enhancing balance and preventing falls in these patients [38,39].

Balance impairments leading to falls are a significant symptom of Parkinson’s disease (PD). Telerehabilitation using wearable technology has become a common practice for patients with PD [40,41]. Virtual assessment and treatment are essential for the efficacy of telerehabilitation in this patient population [42]. It is crucial to measure changes in performance over time when continuously and passively monitoring. Previous studies have used wearable technology to measure gait and turning during daily activities, finding a link to falls and cognition [43,44]. However, further research is needed to understand the impact of telerehabilitation on daily life mobility. This will contribute to the development of a comprehensive virtual balance assessment and evidence-based treatments to aid patients with balance impairments.

Gait analysis also allows for the examination of other critical parameters in assessing patients at risk, especially older adults. Numerous studies have investigated its correlation with adverse health outcomes such as disability, dementia, hospitalization, and mortality [45,46,47]. Key spatiotemporal parameters, including gait speed, stride length, and cadence, are often linked to these negative outcomes [48]. Older adults with slower gait and reduced stride length are considered at increased risk. Specifically, a gait speed below 0.8 m/s is a reliable indicator of an elevated disability risk, while a stride length of 0.64 m is a strong predictor of serious events like physical disability, falls, institutionalization, and mortality [49,50].

Gait analysis offers significant potential in young and athletic individuals, too. Some authors have successfully demonstrated in a double-blind study the ability to identify and differentiate individuals with syndesmotic injury from those with isolated lateral ankle ligament injury in cases of chronic lateral ankle instability using a shoe-integrated sensor system [51]. The results were promising, indicating potential improvements in diagnostic accuracy and the possibility of reducing future reliance on radiological methods while simultaneously optimizing treatment plans.

Finally, some authors have attempted to integrate the diagnostic capabilities of footwear sensors, used for gait analysis, with an interventional component aimed at preventing ankle sprains [52,53,54]. In particular, the system proposed by Attia [53] is based on a gait analysis sensor designed to collect kinematic variables of ankle motion through an inertial motion unit. In real time, acceleration and angular velocity around the foot axis are measured using an accelerometer and gyroscope, respectively. Potentially harmful movements that could lead to an ankle sprain are identified and used to trigger a commercial electrical stimulation device positioned over the peroneal muscles. With a response time of 7 milliseconds and the proven effectiveness of this muscle group in preventing such injuries [55], this approach shows promise and potential for further development.

### 3.2. Gait Analysis Assessment for Post-Treatment Evaluation

The use of gait analysis and motion sensors in post-operative evaluation is becoming increasingly widespread, providing surgeons with valuable tools to guide treatment decisions for individual patients. The literature presents various examples of their application. Some authors have described using these technologies to analyze the outcomes of conservative therapies in athletes with foot disorders, evaluating and comparing the effects of different orthoses [56]. Other potential applications include the use of inertial sensors and baropodometric platforms to assess patients who have undergone surgical procedures like total ankle replacement, allowing for the evaluation of how surgeries that significantly impact proprioception affect the gait cycle post-operatively [57]. Additionally, as proposed by Zhao et al., these tools can help identify the most suitable candidates for specific surgical interventions [58]. In their study, they found that excision of the anterior half of the peroneus longus tendon significantly affected specific gait parameters. The strength of this approach lies in its ability to detect subtle differences that may not be noticeable during normal short walks but could become more pronounced under higher physical demands. Therefore, the authors recommended that the decision to sacrifice the anterior half of the peroneus longus tendon as a harvest site for other procedures should be based on the patient’s athletic demands: it may be suitable for those with low athletic requirements but should be approached with caution for sports enthusiasts or athletes. Evaluations like these would have been nearly impossible to objectify in a simple post-operative clinical assessment, whereas the accuracy and extended wearability of these devices now offer increasingly significant potential.

## 4. Data Collection Methods

Data collection using WT follows a strict protocol to ensure accuracy. The positioning of sensors is crucial, as improper placement can result in unreliable data due to placement errors, and the effectiveness of the algorithms used can vary depending on the target cohort and environment [59,60,61]. Generally, placing sensors on the sacrum, lower anterior thigh, middle lateral shank, and heel is recommended, as these locations tend to produce relatively low error rates [62,63]. Several authors have shown good to excellent validity and reliability of measurements such as step length, stance time, and stride length across various sensor placements (e.g., back, shank, foot) [64,65]. While no specific IMU placement has been proven superior, the pelvis is the most commonly used body location [66]. It is also important to note that novel calibration methods have been recently developed to reduce the need for user movements during sensor placement and alignment with human body segments, further enhancing data accuracy [67,68,69,70].

## 5. Different Types of WT Available

**Figure 2:**
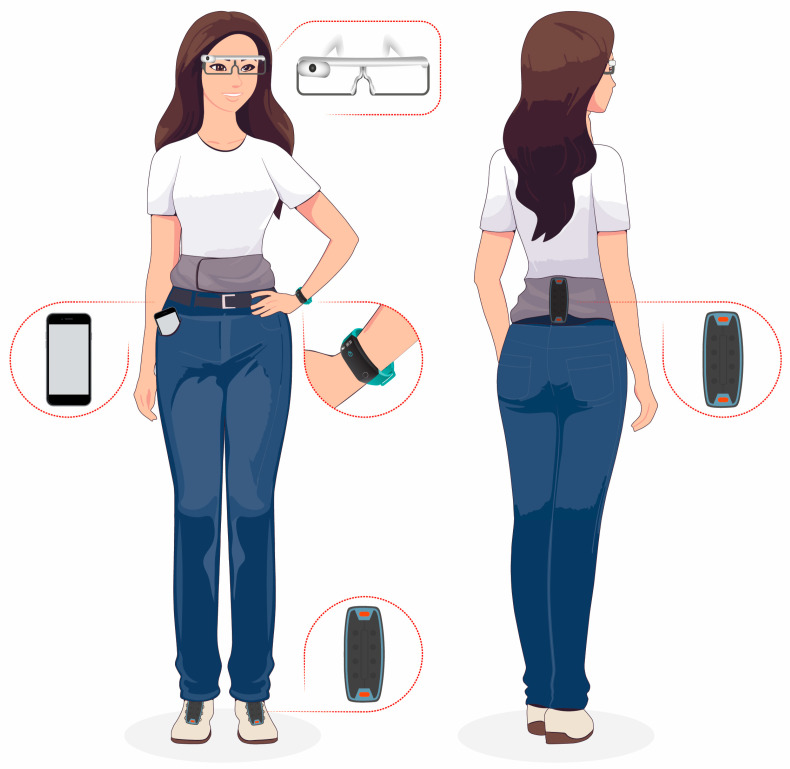
Overview of different wearable technology.

Over the past decade, wearable technology has advanced significantly, becoming lighter, more affordable, and equipped with longer-lasting batteries and increasingly diverse functionalities. Under the umbrella of “WT”, there is a wide range of devices, including shoes, watches, and smart glasses (Figure 2).

A potential issue with wearable sensors is user compliance. However, in the future, this limitation could be mitigated by embedding inertial measurement units (IMUs) into everyday clothing or commonly used devices, making them more user-friendly and seamlessly integrated into daily life [71].

### 5.1. Smartwatches

Smartwatches, often equipped with accelerometers and gyroscopes, have emerged as the most popular form of wearable technology due to their practicality, enjoyment, and user-friendliness. They offer clinicians the ability to monitor patients outside of clinical settings, particularly those who have difficulty accessing clinics due to gait disorders [72]. These devices can track gait speed and count steps, making them potentially useful for identifying physical frailty [73,74,75]. Additionally, smartwatches have demonstrated high accuracy in identifying gait abnormalities, with reported figures of 88.9% accuracy, 90.6% sensitivity, and 86.2% specificity, indicating their promising future in clinical applications [76]. Moreover, their widespread accessibility allows individuals to independently purchase them and to monitor various physiological parameters without requiring clinician intervention. Despite being worn on the upper limbs, smartwatches can effectively and accurately predict step length, swing time, and stance time using machine learning algorithms. Wrist-based IMUs in smartwatches are also employed for gait recognition, freezing of gait detection in Parkinson’s disease, fall detection, and estimating spatiotemporal features [77].

### 5.2. Smartphone

Smartphones have become integral to healthcare and telemedicine, facilitating remote health monitoring of patients [78]. These devices offer continuous, interactive communication from any location, computational power for multimedia applications, and real-time monitoring through wireless sensing technologies. Their role in gait analysis is increasingly prominent across various clinical domains. Smartphones provide a cost-effective means to collect extensive gait data in natural settings. Typically, smartphones are mounted on a sacroiliac belt. However, using looser pants with larger pockets may compromise data quality due to increased movement of the device. Thus, securing the smartphone with a sacroiliac belt is currently regarded as the most reliable approach [79].

### 5.3. Smart Glasses

Smart glasses, though a more recent and less prevalent technology compared to other devices discussed, show significant promise for tracking disease progression in neurological patients [80]. They have demonstrated reliability as an alternative to traditional motion capture systems for gait measurement in healthy adults. Data on head acceleration during walking is particularly valuable for assessing fall risk, underscoring the benefits of head-mounted devices [81,82]. However, the effectiveness of sensor-based gait analysis can be influenced by the wear location, target population, and environment [81]. While smart glasses have proven effective in laboratory and clinical settings for healthy individuals, they face challenges in accurately measuring turning and step parameters [83]. These limitations may stem from discrepancies between the reference sources used by smart glasses and body-worn sensors [84].

### 5.4. Smart Insoles

Sensor-equipped insoles offer versatility and can be applied in various contexts, including sports performance analysis and injury prevention for the foot and ankle. Developing precise and efficient wearable insoles is essential. Several research initiatives have aimed at creating and validating insole systems, such as the “Pedar”, “OpenGo”, and various gait analysis systems like “wi-GAT”, “eSHOE”, and “Medilogic”, which have demonstrated strong validity and reliability [85,86,87]. These devices are now available for a wide range of age groups, including pediatric populations, with models such as “PediaSole” [88]. Recent studies have focused on parameters such as step count, cadence, vertical force, and spatiotemporal gait characteristics. For instance, the Smart Insole can analyze step count, step pace, swing time, and center-of-pressure (COP) shifting velocity. These metrics offer valuable insights into walking balance and fall risk in real-world scenarios. Nevertheless, battery life remains a significant concern for daily use [89].

## 6. Barriers and Limitations

While wearable sensing technologies offer the advantage of continuous gait monitoring beyond laboratory or clinical environments [55,56,69,90,91,92,93], they are not without significant limitations. One primary concern is the limited research on the efficacy and applicability of these technologies for gait and mobility assessment in clinical settings. Despite the growing affordability and accessibility of wearable devices, there is a lack of comprehensive studies evaluating their reliability and validity in real-world applications. This gap in research raises concerns about the accuracy of data collected by these devices [94,95]. For example, certain wearables have been reported to provide inaccurate estimates of activity metrics, such as step counts [96]. Such inaccuracies can compromise the reliability of the data and limit the effectiveness of these devices for precise gait analysis. Additionally, while wearable devices are often praised for their “objective” data due to their physiological and behavioral measurements, the reality is that subjective decisions significantly impact the development and performance of wearable technology and its algorithms. These decisions affect data collection, management, and analysis, which in turn can influence the accuracy and interpretation of the data presented to researchers.

Another limitation is the necessity for a consistent Bluetooth connection for data transmission. Any instability in this connection can lead to data loss, further compromising the reliability of the collected data. Moreover, WT may not be suitable for all populations. Research suggests that individuals with severe motor impairments, those with cognitive impairments, or the elderly may not benefit from wearable devices [97,98]. These groups may face challenges with device usability and data accuracy, underscoring the need for further clinical validation to assess the suitability of WT for these individuals.

A significant area not addressed in this manuscript is the integration of biometric assessment tools, particularly those that leverage sensor-derived measures alongside data analytics and machine learning models as primary endpoints in studies on movement disorders. Future research should delve into the validation and evidence generation surrounding these emerging endpoints, which could enhance both diagnostic precision and patient monitoring.

The integration of WT offers substantial potential to improve patient outcomes and streamline healthcare systems, yet the use of these devices raises notable privacy and data security concerns. Protecting personal health information and ensuring device resilience against cybersecurity threats are crucial steps in harnessing the potential of WT responsibly. While such devices hold the promise of transforming healthcare by offering tailored insights and advancing patient care, privacy risks remain a substantial hurdle. Many wearables and health applications rely on third-party service providers for data storage, processing, and analysis, which complicates oversight of data sharing practices and heightens risks to data privacy.

Although companies adopt agreements to comply with GDPR regulations, privacy breaches remain a possibility, particularly given inconsistencies in GDPR enforcement across EU member states. Addressing these gaps and enforcing stringent, uniform regulations will be critical in fostering public trust and facilitating the responsible integration of WT in healthcare [99].

Overall, while wearable devices have considerable potential, their limitations in data accuracy, usability, and applicability must be carefully considered and addressed through ongoing research and development.

## 7. Conclusions

WT can detect a range of outcome variables, including the ROM of foot and ankle joints, as well as key spatiotemporal gait parameters such as gait speed, stride length, cadence, and balance. These metrics are essential for evaluating post-surgical recovery, the effectiveness of rehabilitation programs, and the risk of adverse health outcomes in both clinical and real-world settings. Proper sensor placement, typically on areas like the sacrum, thigh, shank, and heel, is vital for ensuring reliable measurements, and recent advancements in calibration methods have further improved accuracy. The available types of WT include smartwatches, smartphones, smart glasses, and smart insoles, each offering unique functionalities for monitoring gait and balance, with applications spanning remote health monitoring, fall risk assessment, and sports performance analysis. Future advancements in wearable technology, data analytics, and AI hold the promise of enhancing gait testing in clinical settings by making protocols simpler, more portable, and cost-effective. Nonetheless, the lack of standardized methodologies and procedures remains a significant barrier to widespread adoption.

## Data Availability

Data supporting the reported results can be obtained by writing to lorenzo.brognara2@unibo.it.
